# Supplementary material for: Ongoing monitoring of mindwandering in avoidant grief through cortico-basal-ganglia interactions
Source: Soc Cogn Affect Neurosci. 2018 Dec 6;14(2):163–72. doi: 10.1093/scan/nsy114 (PMC6374603; doi:10.1093/scan/nsy114)
Supplement: Supplementary Data [file nsy114_supp.zip › scan-18-240-File001.docx]

We have responded to the reviewer comment and included quoted sections from the manuscript below. All changes to the manuscript are printed in blue. We have also included a tracked changes and a clean version of the manuscript below.

**Comment 1.1.** The authors have addressed all of my concerns and have improved the clarity of the manuscript in this revision. I would suggest though that the authors consider mentioning that the d-SA network is composed of regions often associated with the default network and salience network (e.g., based on the parcellation of Yeo et al., 2011). Doing so could allow the current findings to be linked to a broader literature on brain networks. I congratulate the authors on an interesting study.

**Response**: Sustained monitoring over mindwandering transpired through interactions between the d-MR basal-ganglia circuit and the d-SA frontotemporoparietal network. The basal ganglia encode motivational salience and habitual responding (Everitt and Robbins, 2005; Berridge, 2007) and receive inputs from across the associative cortex, which allow for the incorporation of new stimuli into the motivational salience framework (Ashby *et al.*, 2010). They interact with the cortical regions seen in the d-SA network such as anterior cingulate and dorsolateral prefrontal cortex, which incorporate higher order motivations and broader goals into salience encoding (Everitt and Robbins, 2005; Grahn *et al.*, 2009). These d-SA regions also form part of the brain’s ventral attention (i.e. attentional salience) and default networks as identified through intrinsic functional connectivity based cortical parcellation (Yeo *et al.*, 2011). Hence, the interacting connectivity between motivational salience, attention and default networks in avoidant grieving may underlie the ongoing conflict over the degree of salience attributed to reminders of the loss and they role they play in capturing attention and altering the default state.
