# Supplementary material for: Ongoing monitoring of mindwandering in avoidant grief through cortico-basal-ganglia interactions
Source: Soc Cogn Affect Neurosci. 2018 Dec 6;14(2):163–72. doi: 10.1093/scan/nsy114 (PMC6374603; doi:10.1093/scan/nsy114)
Supplement: Supplementary Data [file nsy114_supp.zip › scan-18-240-File003.docx]

Title: Ongoing Monitoring of Mindwandering in Avoidant Grief Through Cortico-Basal-Ganglia Interactions

Short Title: Monitoring of Mindwandering in Avoidant Grief

Noam Schneck*^1,2,3^

Tao Tu^3^

Stefan Haufe^4^

George A. Bonanno^5^

Hanga GalfaIvy^2,6^

Kevin Ochsner^7^

J. John Mann^1,2^

Paul Sajda^3,8,9^

1. Division of Molecular Imaging and Neuropathology, New York State Psychiatric Institute, New York, NY

2. Department of Psychiatry, Columbia University, New York, NY

3. Department of Biomedical Engineering, Columbia University, New York, NY

4. Machine Learning Group, Institute of Software Engineering and Theoretical Computer

Science, Technische Universität Berlin, Berlin

5. Department of Clinical Psychology, Teachers College, Columbia University, New York, NY

6. Department of Biostatistics, Columbia University, New York, NY

7. Department of Psychology, Columbia University, New York, NY

8. Department of Radiology, Columbia University, New York, NY

9. Data Science Institute, Columbia University, New York, NY

Word count: #4986

*Address all correspondence and requests for reprints to:

Noam Schneck Ph.D., NYSPI, 1051 Riverside Drive, New York, NY 10032. Tel#: 240-393-9119, email: [schneck@nyspi.columbia.edu](mailto:schneck@nyspi.columbia.edu)

An avoidant grief style is marked by repeated and often unsuccessful attempts to prevent thinking about loss. Prior work shows avoidant grief involves monitoring the external environment in order to avoid reminders of the loss. Here we sought to determine whether avoidant grievers also monitor the internal environment in attempts to minimize conscious awareness of loss-related thoughts. Individuals bereaved of a first-degree relative, spouse or partner within the last 14 months participated in an fMRI study (N=29). We first applied machine learning to train neural patterns for attentional control and representation of the deceased (N=23). The attentional pattern was trained using fMRI data from a modified Stroop task assessing selective attention to reminders of the deceased. The representational pattern was trained using fMRI data from a task presenting pictures and stories of the deceased. We observed spontaneous fluctuations in these processes occurring during a neutral mindwandering fMRI task (N=27). At higher levels of avoidant grieving, activation of attentional control disrupted the relationship between the representational process and thoughts of loss. These findings show that avoidant grief involves attentional control to reduce the likelihood that deceased-related representations reach full conscious awareness.

**Keywords**: MVPA, Grieving, Mindwandering, Basal-Ganglia, Frontotemporoparietal

Avoidant grieving describes a grief style aimed at preventing thoughts of loss from occurring and suppressing them out of consciousness when they do (Shear, 2010; Stroebe and Schut, 2010). Avoidant grievers show more difficult grieving and, paradoxically, more frequent thoughts of loss (Bonanno *et al.*, 2005; Eisma *et al.*, 2013; Eisma *et al.*, 2014; Eisma *et al.*, 2015a). By contrast, those who have less frequent thoughts of loss without exerting effort to do so, have better outcomes (Bonanno *et al.*, 2002). The ongoing effort and failure to prevent thoughts of loss therefore comprises a central dynamic in avoidant grieving.

To prevent thinking of the loss, avoidant grievers are hypervigilant in monitoring the environment for potential reminders of the loss. Monitoring of the external environment has been shown through a faster tendency to push and saccade away from reminders of the deceased (Eisma *et al.*, 2014; Eisma *et al.*, 2015a). Such monitoring underlies an effort to minimize encounter with external reminders of the deceased. However, when mental processing decouples from the environment (i.e. mindwandering) self-generated thoughts arise from the internal environment (Schooler *et al.*, 2011; Smallwood and Schooler, 2015). Avoidant grief may therefore involve monitoring the contents of mindwandering in an attempt to prevent self-generated thoughts of loss. Mindwandering occupies nearly 50% of mental activity (Killingsworth and Gilbert, 2010) and is inherently unpredictable. As a result, continuous monitoring of the contents of mindwandering may occupy a significant amount of the cognitive reserve available to avoidant grievers.

While the external environment contains physical reminders of the loss, the internal environment contains mental representations that may precipitate a thought of loss. Deceased-related mental representations (d-MR) describe the network of information symbolizing the deceased. Thoughts of loss arising during mindwandering can be predicted by activation of a neural deceased-related mental representation network. Moreover, avoidant grievers experience more intense activation of these representations and subsequently more frequent thoughts of loss (Schneck *et al.*, 2017). These findings suggest that mental representations contribute to thoughts of loss and that avoidant grievers attempt to suppress these representations, ironically increasing their salience and frequency. These results accord with general research about the salience increasing effect of attempted suppression (Wegner, 1994). Hence, mental representations of the deceased likely comprise the target for which avoidant grievers scan their internal environment. Once identified, avoidant grievers can attempt to block such representations from reaching consciousness.

This type of attempted control over the mind relies on what has been termed an ironic process, which searches the mental state for undesired or inconsistent content, which the person can then attempt to suppress from reaching consciousness (Wegner, 1994). In grieving, deceased-related selective attention has been shown to identify reminders of the deceased presented externally (Schneck *et al.*, 2018b) and may identify such reminders arising internally. This attention can serve several functions depending on grief-style and context. In *general* grieving, this attention increases immediate engagement with explicitly presented reminders of the deceased (Schneck *et al.*, 2018b) and can be activated independently of conscious loss processing during mindwandering (Schneck *et al.*, 2018a). However, in *avoidant* grieving during mindwandering, selective attention can serve as the ironic process that searches the internal mental space for potential reminders of the deceased to suppress from consciousness (Dehaene and Naccache, 2001). Similar processes of vigilant avoidance, in which people scan the environment for reminders of a threat and then suppress reactions to those reminders, have been identified in repressive coping (Derakshan *et al.*, 2007). We aimed to demonstrate that avoidant grievers employ selective attention (i.e. d-SA) during mindwandering to block arising mental representations (i.e. d-MR) from reaching consciousness.

To test this possibility, we observed ongoing interactions between deceased-related representational (d-MR) and attentional systems (d-SA) during mindwandering. As displayed in Figure 1, our model suggests that during a period of mindwandering, avoidant grievers (darker yellow) employ d-SA (red) to block d-MR (blue) from entering consciousness. Because of the salience-increasing effect of maintained vigilance (Wegner, 1994), avoidant grievers experience more frequent and more intense d-MR (darker blue), paradoxically creating more stress and pressure as d-SA plays a greater role (darker red) in blocking these from reaching consciousness.

To do this, we tracked ongoing neural signatures of d-SA and d-MR during an extended non-emotional sustained attention task designed to promote mindwandering (Smallwood *et al.*, 2004; McVay and Kane, 2013). Tracking of ongoing fluctuations in mental states can be achieved with neural pattern decoding. Neural decoding employs multivariate pattern analysis (MVPA) on a first set of fMRI data to detect a pattern of brain activity associated with a target mental process (i.e. pattern training). This pattern is then applied to a second set of data to predict the occurrence of that mental process (i.e. pattern expression).

Training of the neural pattern for d-SA was implemented using a modified Stroop task. In this task, words reminiscent of the deceased are presented in different colors and subjects report the color of the word as fast as possible. Delays in reporting the color therefore indicate the diversion of selective attention away from word color and towards the meaning of the word (Williams *et al.*, 1996; Holle *et al.*, 1997; Whalen *et al.*, 1998; Algom *et al.*, 2004). While d-SA manifests as longer reaction time in the Stroop task, this task is optimized to identify RT delays linked to initial automatic orientation to reminders of the deceased. Following this initial orientation, d-SA can be used to identify and then suppress reminders from further conscious elaboration.

d-MR comprises the abstract symbol for the deceased that exists independently of a specific perceptual or cognitive modality (Schneck *et al.*, 2017). We sought to delineate a neural model for d-MR incorporating across visual, memory and relational representations of the deceased. We further aimed to ensure that the neural pattern represented the deceased specifically, rather than attachment figures in general, emotional states or demographic features of the deceased. We therefore constructed a task employing pictures, memories and an instruction to imagine being together with a person. These were presented in respect to the deceased, a living-attachment, and a fictional but demographically comparable avatar while assessing ongoing shifts in emotional state. This training task allowed the delineation of a d-MR neural network that incorporated across multiple representational modalities, and was independent of general attachment, demographic and emotional processing.

d-SA and d-MR neural pattern expressions were tracked during two neutral sustained-attention-to-response tasks (SART). One of these was 10-minutes long and contained experience sampling about deceased-related thinking (SART-PROBES) another was 8-minutes long without thought probes (SART). The SART tasks provide relatively non-stimulating environments optimized to promote mindwandering during which we could observe ongoing expression of the d-SA and d-MR patterns (Smallwood *et al.*, 2004; McVay and Kane, 2013). These pattern-expressions provided a proxy for d-SA and d-MR as they transpired during mindwandering (Figure 2). Experience sampling during the SART-PROBES allowed us to determine how avoidant grievers engage d-SA to monitor the contents of mindwandering to prevent d-MR from evolving into self-generated thoughts of loss. Using the SART we were able to explore these processes absent deceased-related cueing.

**Methods**

**Subjects and recruitment**

Twenty-nine people bereaved of a first-degree relative or partner within 14-months participated. Twenty were bereaved by suicide and had been recruited as part of a suicide-bereavement study. Subjects were 18-65 years old, had normal color vision and spoke English as a first language. Recruitment was done through postings on social media websites. All subjects were medically healthy as determined by medical history, examination and standard blood and urine tests. Exclusion criteria were current: bipolar disorder (i.e. manic episode within the past year), substance use disorder (i.e. met criteria within past six months), obsessive-compulsive disorder, and lifetime schizophrenia or schizoaffective disorder assessed with the Structured Clinical Interview for DSM-IV Axis I (SCID I(First *et al.*, 1995)). Subjects taking psychiatric medications were required to be on a stable dose for two weeks prior to scanning. The New York State Psychiatric Institute IRB approved this study and all subjects gave written informed consent.

**Procedure**

Between 3 and 14-months post-loss, subjects underwent a pre-scan interview, an MRI and then a post-scan interview. Interviews occurred within one week of MRI. Grief severity was measured with the Inventory for Complicated Grief (ICG(Prigerson *et al.*, 1997)) and avoidance was measured with the avoidance subscale of the Impact of Events Scale (IES(Zilberg *et al.*, 1982; Baumert *et al.*, 2004)). During the pre-scan interview subjects provided words, pictures and stories relating to the deceased and a relationship matched living control. During the post scan interview subjects completed structured interviews and questionnaires.

**MRI**

MRI acquisition and preprocessing are the same as described in prior work and explained in supplemental information (Schneck *et al.*, 2017; Schneck *et al.*, 2018a; Schneck *et al.*, 2018b).

**Tasks**

**Deceased-Related Selective Attention (d-SA) Task**

During the scan, subjects completed four runs of a cognitive and emotional Stroop task. Each run consisted of four blocks of words: deceased, living, congruent and incongruent. The design for the Stroop task is presented in Figure 3A. Subjects were presented with words and instructed to identify the color of the word font as fast as possible using a right-hand held button box. Training was conducted until subjects reached 100% accuracy and speed of color-button pressing dropped under one second for ten consecutive practice trials. All 15 words were presented for 1.5 seconds and followed by a randomly jittered fixation cross averaging two seconds. In total subjects completed 60 trials per condition. A ten-second fixation cross was presented in between each block. Word presentation and color pairings were randomized within a block and block order was permuted across runs.

**Deceased-related Mental Representations (d-MR) Task**

We used a multi-modal person-processing task to define a neural pattern for d-MR. This task employed three person-conditions (i.e., deceased, living, and demographic control). The living-control accounted for attachment related representations while the demographic control accounted for activity associated with processing demographic features of the deceased. Each person block lasted for 46.5 seconds and comprised three modalities: *picture*, *story*, and *think*. In the *picture modality* two pictures corresponding to the person-condition were displayed for 7.5 seconds each. In the *story* *modality* one of the three stories was presented in successive phrases of three lines with each line being presented for 5 seconds. The stories were alternated across blocks. In the *think* *modality* subjects were instructed to imagine being with the person for 15 seconds. Each modality was separated by a 500ms fixation and each person-condition block was followed by valence (1=Very Sad, 2=Sad, 3=Neutral, 4=Happy, 5=Very Happy) and arousal (1=Very Relaxed, 2=Relaxed, 3=Neutral, 4=Aroused, 5=Very Aroused) probes. Stimulus collection, preprocessing and presentation order are described fully in (Schneck *et al.*, 2017).

**Sustained Attention to Response Task (SART)**

Two SARTs were administered one without thought probes (SART, Figure 3C) and one with thought probes (SART-PROBES, Figure 3D). In the SART, subjects were instructed to press a button every time a number came on screen except for “3”. Numbers were presented on screen for 1.5 seconds with an inter-trial jitter averaging 2 seconds. The number 3 was presented 11% of the time to ensure subjects remained engaged in the task. The SART presented trials continuously for 8 minutes. Following this task retrospective questions were presented about deceased-related thinking occurring during the SART. The SART-PROBES presented trials in blocks of 25-35 seconds. Following each block, thought probes were presented as follows: A) Did you think about *name of deceased* during the past block (Yes/No)? B) Did you think about *name of living control* during the past block (Yes/No)? C) Did you think about yourself during the past block (Yes/No)? Subjects completed 16 blocks. The SARTs were incorporated after the start of the study and therefore two subjects did not perform them.

**Analyses**

This study involved three goals. **1.** Identify neural patterns for d-SA and d-MR based on MVPA analyses of Stroop and Mental Representations fMRI tasks. **2.** Track the continuous expression of d-SA and d-MR patterns during the SARTs**.** **3.** Determine the relationship between avoidant grief style and engagement of d-SA to reduce the relationship between d-MRs and thoughts of loss.

**1. Identifying Neural Patterns for d-SA and d-MR**

**1.1. Feature Selection Overview**

Prior to learning a multivariate pattern for d-SA and d-MR, we used univariate analyses to identify voxels linked to each psychological process. These analyses were performed to limit the voxel input for MVPA and reduce the risk of overfitting and to increase the likeliness that the voxels used for MVPA corresponded to the psychological processes of interest (i.e., d-SA or d-MR). Further explanation is presented in supplemental materials.

**1.2. Feature Selection for d-MR**

Stories and pictures of the deceased from the d-MR task were used to identify a set of voxels involved deceased-related mental representation. This was done through separate univariate t-tests identifying 1) voxels associated with pictures of the deceased *vs*. pictures of both the living-and demographic control attachment and 2) voxels associated with stories of the deceased *vs.* stories of both controls. Both t-tests controlled for self-reported valence and arousal. No significant activation was related to the think instruction and so this was left out of subsequent analyses. To identify a multi-modal set of voxels activated for both pictures AND stories a conjunction analysis was employed on the results of the separate picture and story t-tests (Nichols *et al.*, 2005). The conjunction analysis was thresholded at voxel-p<0.001, cluster–p<0.1. The lenient cluster threshold was used due to the stringency of conjunction analyses and the fact that this analysis was used only for feature selection.

**1.3. Feature Selection for d-SA**

Response time to deceased-related words on the Stroop task was used to identify voxels involved in d-SA. To control for potential confounds of button pressing, sustained attention and motor processing we contrasted with voxels associated with reaction time to congruent words (Deceased-related (BOLD X RT)-Congruent (BOLD X RT)). To ensure that neural activity reflected attentional processing rather than neural reaction to the substantial semantic differences between deceased-related and color-congruent words, a trial level on/off regressor was included. We have previously shown that the process of d-SA likely comprises a subset within a broader process of attachment related attention(Schneck *et al.*, 2018b). As a result, the contrast of deceased-related *vs.* congruent, rather than deceased-related v*s*. living words was used.

A standard hierarchical mixed effects model was employed in FSL to identify voxels whose correlation with reaction time was greater for deceased *vs.* congruent words (Deceased-related (BOLD X RT)-Congruent (BOLD X RT)). This approach rather than a t-test was used to better account for subject level variability in RT to each trial. For this analysis we employed a threshold of voxel-p<0.01 and cluster corrected p<0.05. This threshold was used because d-SA is conceptualized as a relatively broad process that overlaps with attachment related attention and therefore we sought to an inclusive mask of voxels for the sake of incorporating more information into the subsequent MVPA.

**1.4. Pattern Training**

MVPA was applied to the d-MR and d-SA task neural data. In each case MVPA predicted the occurrence of the target psychological process (i.e. d-MR or d-SA) based on neural activity within the feature mask. The neural pattern comprises the relationships between voxels within the feature mask that optimally predict the target process using the labels provided by the tasks (i.e. response time to deceased-related Stroop words for d-SA; pictures and stories of the deceased for d-MR). Across multiple iterations of the prediction, a weighting matrix (*W*) is applied to the neural data, to optimize prediction. Full details of the model training are included in the supplemental information.

**2. Tracking of d-SA and d-MR Pattern Expression during SARTs**

The weighting matrices (*W*) identified in the pattern training steps were applied to the SART datasets. In addition to standard preprocessing, 4D time-series of SART and SART-PROBES data were registered to standard space and motion effects were regressed out using standard FSL 6-degree motion regressors. Each SART time series was also standardized by its own mean and standard deviation.

Pattern expression was estimated by applying the d-SA and d-MR neural patterns (i.e. *W’s)* to the SART and SART-PROBES fMRI data. Model application entails voxel-wise multiplication of the *W’s* in the preselected feature masks with the values for the new BOLD data, followed by a linear summation across voxels. This produces a TR-by-TR model output of the d-SA and d-MR pattern expressions.

After generating the TR-by-TR pattern expression for both d-MR and d-SA as manifested during the SART-PROBES, we calculated blockwise averages of d-SA and d-MR pattern output for each of the 16 blocks. To account for the hemodynamic response delay we applied the model starting at the fourth TR following each probes period and into the second TR into the next probes period. For the SART, the average was calculated for the whole task, starting four TRs after the beginning of the task and continuing two TRs past its conclusion.

**3. Predicting Thoughts of the Deceased During SART-PROBES**

The prior step provided continuous neural proxies of d-SA and d-MR occurring during the SART and SART PROBES. We now aimed to test the relationship between avoidant grief style and the interactive competitive relationship between d-SA and d-MR in relationship to thoughts of loss.

A mixed-effects logistic regression was implemented in R 2.15.13 (Team 2012) to predict self-reported deceased-related thinking during the SART-PROBES from a full factorial model of: d-SA, d-MR and avoidance. This is a longitudinal model identifying predictors of deceased-related thoughts based on d-SA and d-MR expression occurring in the block immediately prior to that self-report across al 16 blocks per-subject. Subject level averages were modeled out as a random intercept. The model included all possible 2-way interactions as well. Blocks of SART-PROBES trials with errors (i.e. commissions or omissions) were excluded from this analysis, because of the potential effects of errors on self-awareness during a task that asks people to retrospect on their thinking.

**Predicting Post-Task Reports of Thoughts of the Deceased Following SART**

We next sought to determine the roles of avoidance, and d-SA and d-MR pattern expression during the SART in predicting post-SART reports of thoughts of the deceased occurring during that time. It was not possible to test interactive relationships between these variables on this dataset because each subject produces only one report of deceased-related thinking summarizing the whole SART time period as compared to sixteen responses per-subject for the SART-PROBES. As a result, we conducted a multiple linear regression investigating subject level avoidance, and subject level averages for pattern expression of d-SA and d-MR during the SART as predictors of post-task reports of thoughts of the deceased.

**Results**

Table 1 describes demographic and clinical characteristics of the sample. ICG mean score of 26.14 (SD=12.84) indicates generally severe grief, although scores had a wide range (1-50). Thoughts of loss occurring on the SART-PROBES correlated with time elapsed since loss, younger age, current depression severity and higher avoidance (Table 1).

**Feature Selection**

For both training tasks, univariate feature selections identified voxel maps associated respectively with d-SA and d-MR. In the Stroop task, BOLD activation in a frontotemporoparietal network correlated with slower responses to deceased-related trials (Figure 4, Table S1, Figure S1). This analysis controlled for semantic word processing and attention to color congruent words. Engagement of these regions in slower *vs.* faster responses to deceased-related trials therefore indicates their involvement in attention to the deceased (i.e. d-SA). We have previously presented the results for the d-MR feature mask and here display it only to identify the anatomical relationship with the d-SA feature mask (Schneck *et al.*, 2017). Voxels in the bilateral basal ganglia, left orbital frontal cortex and insula were associated with deceased-related blocks as compared to control blocks (Figure 4).

**Pattern Training**

For both d-SA and d-MR, pattern-training algorithms were conducted within preselected feature masks generated by the univariate analyses (Figure 4). As previously reported for d-MR (Schneck *et al.* 2017) the multivariate logistic regression model achieved significant cross-validated average out-of-sample classification accuracy of deceased-related pictures and stories (p<0.01) and maximum accuracy achieved was AUC=0.63. For d-SA the MVPA regression model significantly predicted response time to deceased-related words (p=10^-3^). These values are likely inflated due to the pre-selection provided by the univariate analysis. We were not concerned about circularity in this case because the goal of the MVPA analyses was only to identify the corresponding weighting matrices to be used for neural decoding and not to estimate effect sizes or p-values for the training tasks.

**Pattern Expression During SART-PROBES**

Two subjects did not complete the SART-PROBES due to timing limitations. On the SART-PROBES there were a total of 275 error-free blocks, out of which 85 (30%) contained reports of thoughts of loss. There was no significant relationship between errors and thoughts of the deceased (Table 1).

Neural patterns for d-SA and d-MR were applied to the SART and SART-PROBES fMRI data to produce ongoing proxies of d-SA and d-MR during these time periods (Figure 2C and 2D). Pattern expression outputs for the d-MR and d-SA neural patterns were inspected for outliers falling outside the interquartile range. Five outlier values were identified for d-MR and one for d-SA, these were winsorized (i.e. censored down to the nearest value within the interquartile range). d-MR pattern expression was not significantly related to d-SA expression or errors during either the SART (F_350_ =.55, p=.45, F_329_ =.97, p=.32) or the SART PROBES (F_265_ =.26, p=.61, F_402_ =.33, p=.56).

**Predicting Thoughts of the Deceased During SART-PROBES**

The three-way interaction of d-SA, d-MR and avoidance significantly predicted thoughts of loss. Specifically, the multiplicative term combining all 3 variables predicted the odds of a thought of loss as 1/10 that of the odds predicted by the lower level 2-way interactions (Odds ratio (OR; for one standard deviation change in predictor)=.09, Table 2). Meaning, that for high avoidant subjects, as d-SA increased the prediction of thoughts of loss from d-MR was less than 1/10 the odds as predicted by any of the two-way interaction combinations. The combination of avoidance, d-SA and d-MR therefore served as an accurate predictor of the lack of thinking about the loss as compared to other models. This effect was maintained when controlling for age, loss-type, time-since-loss and depression severity (*B=*-21.41, p=.003, OR=.08, 95%CI: 0.02-0.38).

To parse this interaction, a median split was used to create high (n=17) and low (n=10) avoidance groups. For subjects in the high avoidance group, the relationship between d-MR and thoughts of loss was moderated by d-SA. Meaning, on blocks with high d-SA expression, increasing d-MR expression was less likely to result in a thought of loss (Figure 5B, Blue Line). On blocks with lower d-SA expression, as d-MR expression increased the odds of a thought of loss increased as well (Figure 5B, Red Line). Specifically, as d-SA increased, the odds of a thought of loss predicted by d-MR was approximately 1/3^rd^ the odds that of a thought of loss predicted by d-SA and d-MR alone (OR=.35, Table 2). Meaning that as d-SA increased the relationship between d-MR and a thought of loss decreased in the high avoidance group. In the low avoidance group, the opposite was found. As d-SA expression increased the odds that d-MR would predict a thought of loss also increased (OR=2.14, Figure 5A, Table 2).

**Specificity of 3-way Interaction to d-SA and d-MR Neural Patterns**

To test the extent to which this three-way interaction was specific to the neural pattern models identified for d-SA and d-MR in the training tasks, rather than being explained by arbitrary fluctuations in neural data occurring during the SART-PROBES, we extracted the average BOLD signal in both the d-SA and d-MR feature masks across the same time period during the SART-PROBES for which the neural patterns were applied (i.e. from 4 TRs after each probe period until 2 TRs into the next probes period). This produced the output for each TR corresponding to average BOLD signal in the d-SA feature mask and one corresponding to the d-MR feature mask. We then used these outputs to calculate the three-way interaction now predicting thoughts of loss from average d-MR feature mask BOLD signal, average d-SA feature mask BOLD signal and subject level avoidance. As expected this 3-way interaction was not significantly predictive of thoughts of loss (p=.17)

**Predicting Post-Task Reports of Thoughts of the Deceased on the SART**

On the SART, no thought probes were presented in-task but subjects did indicate deceased-related thinking at the end of the task. While controlling for both d-SA and avoidance, reduced d-MR during the SART predicted higher post-task reports of thoughts of loss occurring during the task, accounting independently for 31% of variance in post-task reported thoughts of loss (**d-MR**: B(SE)_26_*=*-71,46(24.44), t=-2.92, *p*=.009, 95%CI=-122.82 to -20.1 **d-SA**: B (SE)_26_*=*2.40(2.29), t=1.04, *p*=..31, 95%CI=-2.4 to 7.2, **IES-A:** B(SE)_26_*=*.33(.25), t=1.30, *p*=.21, 95%CI= -.2 to .86).

**Discussion**

This is the first study to show that avoidant grievers monitor the contents of their mindwandering. This monitoring was linked to a reduced likeliness that mental representations of the deceased, arising during mindwandering, would lead to a conscious thought of loss. We build on prior research demonstrating avoidant monitoring of the *external* environment to avoid encounter with reminders of loss (Eisma *et al.*, 2014; Eisma *et al.*, 2015a). Ongoing monitoring over the contents of mindwandering in addition to the external environment may detract from engagement in other life activities and contribute to the negative outcomes linked to this grief style.

Despite attempts to monitor mindwandering and avoid thoughts of loss, avoidant grievers still displayed more intrusive thoughts of loss *overall* on the task (Table 1). This finding accords with general research on avoidant grieving and research showing that attempted thought suppression ironically results in greater frequency of the target thought (Wegner, 1994; Eisma *et al.*, 2013; Eisma *et al.*, 2015b). Our findings suggest a mechanism by which this paradoxical effect occurs. During periods of mindwandering when mental representations of the deceased were low and deceased-related selective attention high, avoidant grievers were more likely to experience thoughts of loss (Figure 5B). Hence, the monitoring itself (i.e. d-SA) in the absence of a target to be suppressed (i.e. d-MR) may lead to thoughts of loss and contribute to the paradoxical effects of increased deceased-related thinking in avoidant grieving. Future longitudinal studies can also test whether this internal monitoring predicts worse grief outcomes.

The present study characterizes several states of deceased-related processing in avoidant grief (Figure 6). 1) **Task focus**. This state is most common amongst non-avoidant grievers who, when engaged in an external task, evidence little effect of d-MRs on actual conscious thoughts of loss. 2) **Suppression**: In this state activated d-MRs are suppressed from consciousness by simultaneous d-SA, 3) **Intrusion**: During intrusion d-SA is low and ongoing activated d-MRs correlate with spontaneous thoughts of loss. Avoidant people exhibit a high degree of intrusion and suppression, which may both, contribute to the poor clinical outcomes associated with this coping style.

Sustained monitoring over mindwandering transpired through interactions between the d-MR basal-ganglia circuit and the d-SA frontotemporoparietal network. The basal ganglia encode motivational salience and habitual responding (Everitt and Robbins, 2005; Berridge, 2007) and receive inputs from across the associative cortex, which allow for the incorporation of new stimuli into the motivational salience framework (Ashby *et al.*, 2010). They interact with the cortical regions seen in the d-SA network such as anterior cingulate and dorsolateral prefrontal cortex, which incorporate higher order motivations and broader goals into salience encoding (Everitt and Robbins, 2005; Grahn *et al.*, 2009). These d-SA regions also form part of the brain’s ventral attention (i.e. attentional salience) and default networks as identified through intrinsic functional connectivity based cortical parcellation (Yeo *et al.*, 2011). Hence, the interacting connectivity between motivational salience, attention and default networks in avoidant grieving may underlie the ongoing conflict over the degree of salience attributed to reminders of the loss and they role they play in capturing attention and altering the default state.

Our results indicate that employment of d-SA to monitor d-MR is context-dependent. In the absence of thought probes, during the SART, less d-MR engagement, irrespective of avoidance-style and d-SA, predicted higher post-task reports of thoughts of loss. These findings suggest that the role of d-SA in directing conscious processing towards or away from the loss may operate specifically in contexts that emphasize meta-cognition about deceased-related thinking. However, due to limited sample size this analysis was less powered than the SART-PROBES analysis and therefore this conclusion is mentioned as a suggestion only.

Methodologically, this work builds on studies using neural decoding to track mental processes unfolding naturalistically. Prior work has used neural decoding to track negative mood and other emotional states as they transpired over time (Tusche *et al.*, 2014; Kragel *et al.*, 2016). We build on this work by tracking ongoing interactions between mental states as they occur spontaneously.

**Limitations and Future Studies**

This study grouped together grieving a loss by suicide and non-suicide. While we controlled for in analyses it is possible that grouping of high and low avoidance subjects by loss type may have influenced findings. Future studies can recruit specifically across levels of avoidance in the same type of bereavement. Furthermore, incorporation of physiological monitoring during the SART may also provide more information about thought processes occurring during this time.

A central neural correlate of mindwandering is connectivity in the default network (DN)(Andrews-Hanna *et al.*, 2014). A future study can assess the relationship between the mental processes of selective attention and mental representation investigated here and ongoing DN connectivity. It is likely that as vigilant monitoring over mental representations is engaged DN connectivity would decrease as the mind becomes involved in the task of vigilance.

**Conclusions**

Avoidant grievers monitored mindwandering in a way that disrupted the potential occurrence of self-generated thoughts of loss. This monitoring manifested through the continuous application of an attentional network over a representational network during mindwandering, which was linked to a reduced relationship between the latter and self-generated thoughts of loss. The taxing effects of maintaining constant vigilance over ever-evolving contents of mindwandering may contribute to the poor outcomes linked to deliberate grief avoidance.

**Acknowledgements**

This study was generously funded by a Distinguished Investigator Grant (DIG-0-163-12) (JJM) and a Young Investigator Grant (YIG-0-215-13) (NS) from the American Foundation for Suicide Prevention. NS was supported by a National Institute of Mental Health T32 Training Grant in Anxiety and Related Disorders.

**Financial Disclosures**

Dr. Mann receives royalties for commercial use of the C-SSRS from the Research Foundation for Mental Hygiene and has stock options in Qualitas Health, a start- up developing a PUFA supplement. Dr. Sajda is the majority owner and Chairman of the Board of Neuromatters LLC, a brain computer interface neuromarketing and gaming company. Dr. Galfalvy’s family owns stock in Illumina, Inc. Dr. Schneck, Dr. Bonnano, Dr. Ochsner, Dr. Haufe and Mr. Tu have no conflicts of interests to declare.

**References**

Algom, D., Chajut, E. & Lev, S. (2004) 'A rational look at the emotional stroop phenomenon: a generic slowdown, not a stroop effect', *J Exp Psychol Gen*, **133**(3), pp. 323-338.

Andrews-Hanna, J. R., Smallwood, J. & Spreng, R. N. (2014) 'The default network and self-generated thought: component processes, dynamic control, and clinical relevance', *Ann N Y Acad Sci*, **1316**, pp. 29-52.

Ashby, F. G., Turner, B. O. & Horvitz, J. C. (2010) 'Cortical and basal ganglia contributions to habit learning and automaticity', *Trends Cogn Sci*, **14**(5), pp. 208-215.

Baumert, J., Simon, H., Gundel, H., Schmitt, C. & Ladwig, K. H. (2004) 'The Impact of Event Scale--Revised: evaluation of the subscales and correlations to psychophysiological startle response patterns in survivors of a life-threatening cardiac event: an analysis of 129 patients with an implanted cardioverter defibrillator', *J Affect Disord*, **82**(1), pp. 29-41.

Berridge, K. C. (2007) 'The debate over dopamine's role in reward: the case for incentive salience', *Psychopharmacology (Berl)*, **191**(3), pp. 391-431.

Bonanno, G. A., Papa, A., Lalande, K., Zhang, N. & Noll, J. G. (2005) 'Grief processing and deliberate grief avoidance: a prospective comparison of bereaved spouses and parents in the United States and the People's Republic of China', *J Consult Clin Psychol*, **73**(1), pp. 86-98.

Bonanno, G. A., Wortman, C. B., Lehman, D. R., Tweed, R. G., Haring, M., Sonnega, J., Carr, D. & Nesse, R. M. (2002) 'Resilience to loss and chronic grief: a prospective study from preloss to 18-months postloss', *J Pers Soc Psychol*, **83**(5), pp. 1150-1164.

Dehaene, S. & Naccache, L. (2001) 'Towards a cognitive neuroscience of consciousness: basic evidence and a workspace framework', *Cognition*, **79**(1-2), pp. 1-37.

Derakshan, N., Eysenck, M. W. & Myers, L. B. (2007) 'Emotional information processing in repressors: The vigilance-avoidance theory', *Cognition & Emotion*, **21**(8), pp. 1585-1614.

Eisma, M. C., Rinck, M., Stroebe, M. S., Schut, H. A., Boelen, P. A., Stroebe, W. & van den Bout, J. (2015a) 'Rumination and implicit avoidance following bereavement: an approach avoidance task investigation', *J Behav Ther Exp Psychiatry*, **47**, pp. 84-91.

Eisma, M. C., Schut, H. A., Stroebe, M. S., Boelen, P. A., van den Bout, J. & Stroebe, W. (2015b) 'Adaptive and maladaptive rumination after loss: A three-wave longitudinal study', *Br J Clin Psychol*, **54**(2), pp. 163-180.

Eisma, M. C., Schut, H. A., Stroebe, M. S., van den Bout, J., Stroebe, W. & Boelen, P. A. (2014) 'Is rumination after bereavement linked with loss avoidance? Evidence from eye-tracking', *PLoS One*, **9**(8), p. e104980.

Eisma, M. C., Stroebe, M. S., Schut, H. A., Stroebe, W., Boelen, P. A. & van den Bout, J. (2013) 'Avoidance processes mediate the relationship between rumination and symptoms of complicated grief and depression following loss', *J Abnorm Psychol*, **122**(4), pp. 961-970.

Everitt, B. J. & Robbins, T. W. (2005) 'Neural systems of reinforcement for drug addiction: from actions to habits to compulsion', *Nat Neurosci*, **8**(11), pp. 1481-1489.

First, M., Spitzer, R., Gibbon, M. & Williams, J. (1995) *Structured Clinical Interview for DSM-IV Axis I Disorders (SCID-I/P, Version 2.0)*, New York, Biometrics Research Dept., New York State Psychiatric Institute.

Grahn, J. A., Parkinson, J. A. & Owen, A. M. (2009) 'The role of the basal ganglia in learning and memory: neuropsychological studies', *Behav Brain Res*, **199**(1), pp. 53-60.

Holle, C., Neely, J. H. & Heimberg, R. G. (1997) 'The effects of blocked versus random presentation and semantic relatedness of stimulus words on response to a modified Stroop task among social phobics', *Cognitive Therapy and Research*, **21**(6), pp. 681-697.

Killingsworth, M. A. & Gilbert, D. T. (2010) 'A wandering mind is an unhappy mind', *Science*, **330**(6006), p. 932.

Kragel, P. A., Knodt, A. R., Hariri, A. R. & LaBar, K. S. (2016) 'Decoding Spontaneous Emotional States in the Human Brain', *PLoS Biol*, **14**(9), p. e2000106.

McVay, J. C. & Kane, M. J. (2013) 'Dispatching the wandering mind? Toward a laboratory method for cuing "spontaneous" off-task thought', *Front Psychol*, **4**, p. 570.

Nichols, T., Brett, M., Andersson, J., Wager, T. & Poline, J. B. (2005) 'Valid conjunction inference with the minimum statistic', *Neuroimage*, **25**(3), pp. 653-660.

Prigerson, H. G., Bierhals, A. J., Kasl, S. V., Reynolds, C. F., Shear, M. K., Day, N., Beery, L. C., Newsom, J. T. & Jacobs, S. (1997) 'Traumatic grief as a risk factor for mental and physical morbidity', *American Journal of Psychiatry*, **154**(5), pp. 616-623.

Schneck, N., Haufe, S., Tu, T., Bonanno, G. A., Ochsner, K., Sajda, P. & Mann, J. J. (2017) 'Tracking Deceased-Related Thinking with Neural Pattern Decoding of a Cortical-Basal Ganglia Circuit', *Biol Psychiatry Cogn Neurosci Neuroimaging*, **2**(5), pp. 421-429.

Schneck, N., Tu, T., Bonanno, G. A., Shear, M. K., Sajda, P. & Mann, J. J. (2018a) 'Self-generated Unconscious Processing of Loss Linked to Less Severe Grieving', *Biol Psychiatry Cogn Neurosci Neuroimaging*.

Schneck, N., Tu, T., Michel, C., Bonanno, G. A., Sajda, P. & Mann, J. J. (2018b) 'Attentional Bias to Reminders of the Deceased as Compared to a Living Attachment in Grieving ', *Biological Psychiatry: Cognitive Neuroscience and Neuroimaging*, **3**(2), pp. 107-115.

Schooler, J. W., Smallwood, J., Christoff, K., Handy, T. C., Reichle, E. D. & Sayette, M. A. (2011) 'Meta-awareness, perceptual decoupling and the wandering mind', *Trends Cogn Sci*, **15**(7), pp. 319-326.

Shear, M. K. (2010) 'Exploring the role of experiential avoidance from the perspective of attachment theory and the dual process model', *Omega (Westport)*, **61**(4), pp. 357-369.

Smallwood, J., Davies, J. B., Heim, D., Finnigan, F., Sudberry, M., O'Connor, R. & Obonsawin, M. (2004) 'Subjective experience and the attentional lapse: task engagement and disengagement during sustained attention', *Conscious Cogn*, **13**(4), pp. 657-690.

Smallwood, J. & Schooler, J. W. (2015) 'The science of mind wandering: empirically navigating the stream of consciousness', *Annu Rev Psychol*, **66**, pp. 487-518.

Stroebe, M. & Schut, H. (2010) 'The dual process model of coping with bereavement: a decade on', *Omega (Westport)*, **61**(4), pp. 273-289.

Tusche, A., Smallwood, J., Bernhardt, B. C. & Singer, T. (2014) 'Classifying the wandering mind: revealing the affective content of thoughts during task-free rest periods', *Neuroimage*, **97**, pp. 107-116.

Wegner, D. M. (1994) 'Ironic processes of mental control', *Psychol Rev*, **101**(1), pp. 34-52.

Whalen, P. J., Bush, G., McNally, R. J., Wilhelm, S., McInerney, S. C., Jenike, M. A. & Rauch, S. L. (1998) 'The emotional counting Stroop paradigm: a functional magnetic resonance imaging probe of the anterior cingulate affective division', *Biol Psychiatry*, **44**(12), pp. 1219-1228.

Williams, J. M., Mathews, A. & MacLeod, C. (1996) 'The emotional Stroop task and psychopathology', *Psychol Bull*, **120**(1), pp. 3-24.

Yeo, B. T., Krienen, F. M., Sepulcre, J., Sabuncu, M. R., Lashkari, D., Hollinshead, M., Roffman, J. L., Smoller, J. W., Zollei, L., Polimeni, J. R., Fischl, B., Liu, H. & Buckner, R. L. (2011) 'The organization of the human cerebral cortex estimated by intrinsic functional connectivity', *J Neurophysiol*, **106**(3), pp. 1125-1165.

Zilberg, N. J., Weiss, D. S. & Horowitz, M. J. (1982) 'Impact of Event Scale: a cross-validation study and some empirical evidence supporting a conceptual model of stress response syndromes', *J Consult Clin Psychol*, **50**(3), pp. 407-414.

**Table 1. Clinical and Demographic Characteristics**

| Demographic and Clinical Variables | M (SD) | Correlation with Thoughts of Loss on SART-PROBES | | |
| --- | --- | --- | --- | --- |
| Age | 44.06 (13.65) | -.43** | | |
| Months Since Loss | 8.06 (4.79) | .51** | | |
| CES-D | 1.63 (.43) | .59** | | |
| IES-A | 1.71 (.59) | .47* | | |
| Education years | 16.43 (1.9) | -.35 | | |
| Medication Use | N=10 (34%) | .25 | | |
| Errors on SART-PROBES | 7.52 (5.35) | .12 | | |
| Thoughts of Loss on SART^1^ | 1.67 (.75) | .44* | | |
| Thoughts of Loss on SART-PROBES^2^ | .31 (.34) | 1 | | |
| Males | N=6 |  |  |  |

*Correlation significant at p<0.05.

** Correlation significant at p<0.01.

^1^ Calculated as average response to probes about thoughts of loss occurring during beginning middle and end of SART. Responses scored on a scale of: 1- “Did not think about deceased” to 4- “Thought about deceased consistently”.

^2^ Calculated as percentage of correct blocks during SART-PROBES in which thoughts of loss were reported.

ICG= Inventory for Complicated Grief, CES-D= Center for Epidemiological Studies-Depression, IES-A= Impact of Event Scale- Avoidance.

**Table 2. Predicting Thoughts of Loss from D-MR, D-SA and Avoidance**

|  | *B*^1^ | OR^2^ | 95% CI | *p* |
| --- | --- | --- | --- | --- |
| **3-Way Interaction** |  |  |  |  |
| Deceased-related Mental Representations (d-MR) | .33 (.66) | 1.45 | .73-2.68 | *.39* |
| Deceased-related Selective Attention (d-SA) | .72 (1.17) | 2.06 | 1.17-4.09 | *.27* |
| Avoidant Grief Style (IES-A) | 1.0 (.54) | 2.73 | .94-7.94 | *.07* |
| d-MR x d-SA | 2.21 (.84) | 9.11 | 1.92-43.18 | *.93* |
| d-MR x IES-A | -.25 (.71) | .77 | .21-2.90 | *.43* |
| d-SA X IES-A | 1.11 (.73) | 3.06 | .73-12.88 | *.37* |
| d-SA x d-MR x IES-A | -2.37 (.82) | .09 | .02-.47 | ***.004*** |
| **2-Way Interaction-High Avoidant Grief Style** |  |  |  |  |
| d-MR | -.13 (.31) | .88 | .48-1.60 | *.66* |
| d-SA | .68 (.29) | 1.96 | 1.1-3.5 | ***.02*** |
| d-MR x d-SA | -1.04 (.38) | .35 | .17-.75 | ***.006*** |
| **2-Way Interaction- Low Avoidant Grief Style** |  |  |  |  |
| d-MR | .3 (.28) | 1.36 | .78-2.38 | *.26* |
| d-SA | .11 (.34) | 1.12 | .58-2.2 | *.73* |
| d-MR x d-SA | .76 (.31) | 2.14 | 1.15-3.99 | ***.01*** |

^1^ Logistic regression coefficients are reported for one standard deviation increase in the predictor. ^2^ OR= Odds ratio for one standard deviation change in predictor.

**Table 2**. Results from mixed effect logistic regressions predicting thoughts of loss during the SART-PROBES from ongoing interactions between d-SA and d-MR as a function of avoidant grief style. Results are presented as a 3-way interaction for the entire sample spanning levels of avoidant grief style as well as a 2-way interaction between d-MR and d-SA at high and low levels of avoidant grief style.

**Figure Captions**

**Figure 1. Mental Processes Underlying Self-Generated Thoughts of Loss During Mindwandering.** This figure displays a model by which individuals with a more avoidant grief style (i.e. darker yellow) experience more frequent and intense activation of mental representations of the deceased (i.e. darker blue arrows). In the context of this activation, the engagement of selective attention (i.e. darker red) blocks representations from reaching conscious awareness. When avoidant people fail to engage selective attention (i.e. lighter red), the activated mental representations do reach conscious awareness (i.e. arrows entering consciousness).

**Figure 2. Continuous Pattern Expression:** Neural patterns for d-SA and d-MR were respectively derived from the Stroop and Mental Representations tasks using MVPA. Neural decoding was then applied by tracking the expressions of these patterns during both SART tasks. These expressions served as a proxy for the fluctuations of d-SA and d-MR as they unfolded during the neutral SART tasks.

**Figure 3. Pattern Training and Expression fMRI Tasks:** An emotional Stroop and mental representation task were used to identify neural patterns for deceased-related selective attention and mental representation. Words were presented in the Stroop task in red, green or blue and are written here in black for display only. The expression of these patterns was then tracked during a 10-minute neutral sustained attention task and a second identical task with interspersed thought probes.

**Figure 4. Basal Ganglia-Orbitofrontal and Frontotemporoparietal Feature Masks:**  Feature masks produced by univariate analyses for d-MR (blue) and d-SA (red) were used as input to respective MVPA analyses. For the d-MR feature mask, activation is seen in basal ganglia, orbital frontal cortex and insula (voxel-p <.001). For the d-SA mask, activation is seen in a broad frontotemporoparietal network (voxel-p <.01, cluster-p <.05).

**Figure 5. Predicting Thoughts of Loss from Blockwise Pattern Expression of d-SA and d-MR at High and Low Levels of Avoidance:** This figure displays the predicted probability of a thought of loss based on block-wise d-SA and d-MR output for subjects in the high and low avoidance groups separately. For subjects in the high avoidance group (N=17), d-SA negatively moderated the relationship between d-MR and thoughts of loss (p=.02) Specifically, lower d-SA expression corresponded to a positive relationship between d-MR expression and the probability of a thought of loss occurring on that block (Red line). Conversely, on blocks with higher d-SA expression the relationship between d-MR expression and the probability of a thought of the deceased diminished (Blue Line). For subjects in the low avoidance group (N=10) d-SA increased the relationship between d-MR pattern expression on a given block and thoughts of loss (p=.01).

**Figure 6. Hypothetical Model of Deceased-Related Intrusive Processing**. Three states of deceased-related intrusive processing are defined. During task focus, d-MR is limited and not likely to lead to a thought of loss. During intrusion, d-MR is activated and d-SA is not engaged, resulting in intrusive thoughts of loss. During suppression, d-MR and d-SA are engaged, leading to suppression of activated d-MRs from consciousness. Avoidant grievers evidence more frequent states of intrusion and suppression.
