# Supplementary material for: Ongoing monitoring of mindwandering in avoidant grief through cortico-basal-ganglia interactions
Source: Soc Cogn Affect Neurosci. 2018 Dec 6;14(2):163–72. doi: 10.1093/scan/nsy114 (PMC6374603; doi:10.1093/scan/nsy114)
Supplement: Supplementary Data [file nsy114_supp.zip › scan-18-240-File010.docx]

**MRI Preprocessing**

Preprocessing was carried out using FSL version 6 (FMRIB's Software Library, [www.fmrib.ox.ac.uk/fsl](http://www.fmrib.ox.ac.uk/fsl))(Woolrich et al. 2009). Preprocessing included slice time correction, motion correction, skull stripping and smoothing with a Gaussian kernel of 6mm FWHM. A 120-second high pass filter was applied to the data. All data were corrected for head motion by removing the influence of six motion time courses. Bias field correction was implemented using FSL-FAST for functional and structural images (Zhang et al. 2001). Following preprocessing functional images were registered to structural images with 7-degrees of freedom and then structural images were warped to the standard MNI space using a 12-degree affine registration followed by a non-linear warp implemented in FNIRT (Jenkinson et al. 2002; J.L.R. Andersson 2007). Following the acquisition of the first 23 subjects, the T1 bias correction step failed for two subjects necessitating the removal of this step from preprocessing. To reduce the effect of this noise on the overall model, subsequent subjects were excluded from the pattern-training phase (i.e. Stroop task) and only incorporated in the pattern application phase (SART-PROBES). All analyses were run with and without these subjects and effects were unchanged. All images were registered to the MNI standard space template. All regional delineations are defined according to the Harvard-Oxford atlases.

**Explanation of Univariate Analyses**

Univariate analyses were employed to ensure that the voxels submitted to MVPA corresponded to the specific target psychological processes. This was necessary because MVPA analyses can combine across multiple mental processes represented in diffuse voxel space to predict a given stimulus. For example, presentation of pictures of the deceased may elicit the target process (i.e. d-MR) as well as other processes (i.e. arousal). An MVPA will usually learn the combination of these processes as represented in voxel space that best predicts when the pictures are being shown. While this is useful for optimizing prediction of the pictures being shown, the resultant neural pattern now combines d-MR, the target process, and arousal, a separate process. While it is possible to control for arousal, as we did, the MVPA is still capable of learning a pattern of combination of arousal and d-MR unique to the presentation of deceased-related pictures. Because our primary aim was to develop a neural pattern primarily involved in d-MR that would track with d-MR as it arose in a separate context (i.e. the SART) in which other processes such as arousal may not be linked to d-MR we sought to identify a neural pattern only responsible for the primary mental processes of interest. As such, the first pass, univariate analysis aimed to exclude voxels involved in any mental process outside of the target processes of d-SA or d-MR.

**MVPA**

**d-MR and d-SA Pattern Training.**

For the d-MR prediction, we used l_2_-norm regularized linear logistic regression implemented in Fast Simultaneous Training of Generalized Linear Models (FaSTGLZ^30^). This analysis maximally separates the projected BOLD signal of deceased trials on one hand, and control trials on the other hand. L_2_-norm regularized linear logistic regression optimizes the cost function $\sum_{i=1}^{N} {[y}_{i}\boldsymbol{x}_{i}\boldsymbol{\beta-}N\log(1+e^{\boldsymbol{x}_{i}\boldsymbol{\beta}})]+ \lambda\sum_{j=1}^{K} \beta_{j}$, where $N$ is the number of trials, $K$ is the number of voxels in the mask, $y_{i}$ is the class label of the i-th trial, encoded as +1 for deceased trials, and as -1 for control trials, $\boldsymbol{x}_{i}$ is the vector of BOLD data for the i-th trial, and $\boldsymbol{\beta=}{\boldsymbol{[}\beta_{1},\ldots, \beta_{K}]}^{T}$ are linear regression coefficients mapping BOLD data to class labels. The constant λ implements a tradeoff between model fidelity and model complexity as measured by the sum of squared regression coefficients. λ was optimized using 10x10 fold cross validation in order to maximize the model's prediction accuracy on hold out data. That is, the blocks were randomly split into 10 parts (folds), where nine folds were used to train the models (one for each choice of the regularization constant), and the tenth fold was used to measure the out-of-sample classification accuracy of the model in terms of the area under the receiver operating curve (AUC). Each fold served as holdout set once, and the procedure was repeated ten times for different random splits of the data, giving rise to 100 out-of-sample AUC scores per regularization constant. AUC values were averaged to yield one value per choice of the regularization constant. The entire analysis was repeated for 100 randomly permuted vectors of class labels, which allowed us to test the statistical significance of the obtained classifications against a null distribution. The optimal regularization constant was chosen as the maximizer of this value, and used to train a final model based on the data of all trials.

The analysis for the Stroop trials used a multivariate linear regression to predict reaction time for deceased-related trials on the basis of neural data within the d-SA feature mask. We used an L_2_-norm regularized linear regression model implemented in FaSTGLZ^30^ to predict the deceased related RT using the BOLD activity across multiple voxels selected by the d-SA feature mask. To test the significance of the prediction, we used a permutation procedure where we randomly permuted the RT and calculated the mean squared error (MSE) of the prediction. This procedure was repeated for 1000 times and we obtain an empirical null distribution of the MSE. The significance of the prediction was then determined by comparing the observed MSE against the empirical null distribution.


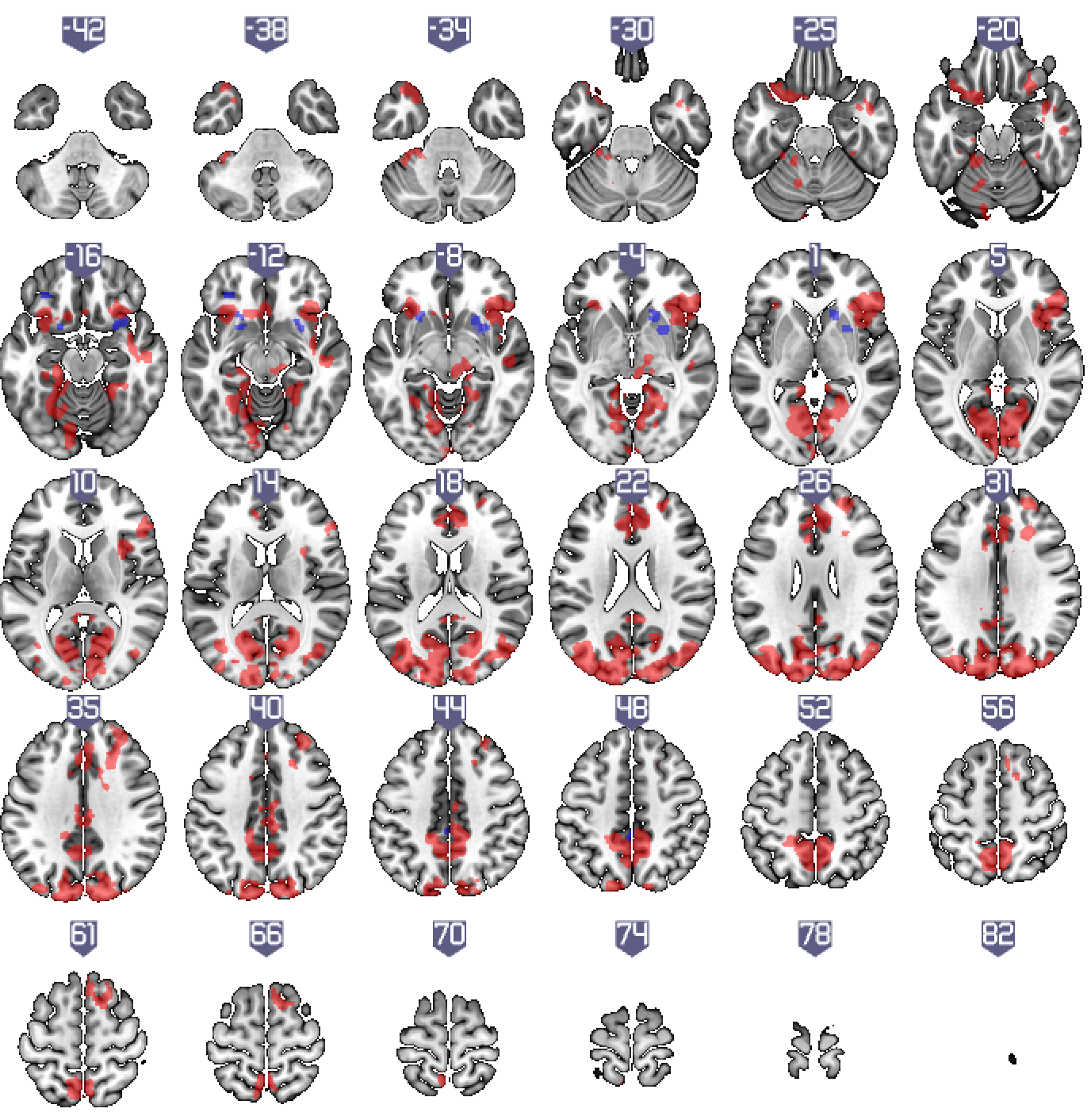


**Figure S1.** Cross-slice image depicting full extent of both d-SA (red) and d-MR (blue) networks

| **Table S1. Clusters associated with deceased-related selective attention (d-SA)** | | | | | |
| --- | --- | --- | --- | --- | --- |
|  | # Voxels | Cluster Center (X,Y,Z) | | | z-score |
| Brain Stem | 158 | 43.46 | 47.72 | 30.39 | 2.66 |
| Central Opercular Cortex | 19 | 24.74 | 67.63 | 38.74 | 2.63 |
| Cingulate Gyrus, anterior division | 440 | 44.58 | 76.14 | 48.54 | 2.60 |
| Cingulate Gyrus, posterior division | 546 | 45.11 | 43.92 | 48.63 | 2.53 |
| Cuneal Cortex | 845 | 45.48 | 23.15 | 50.15 | 2.76 |
| Frontal Operculum Cortex | 187 | 24.00 | 72.66 | 37.04 | 2.71 |
| Frontal Orbital Cortex | 1017 | 43.37 | 73.73 | 29.18 | 2.75 |
| Frontal Pole | 474 | 30.22 | 85.63 | 50.15 | 2.64 |
| Inferior Frontal Gyrus, pars opercularis | 10 | 19.90 | 72.80 | 38.40 | 2.41 |
| Inferior Frontal Gyrus, pars triangularis | 313 | 19.54 | 77.25 | 37.83 | 2.67 |
| Insular Cortex | 450 | 32.31 | 69.87 | 34.02 | 2.65 |
| Intracalcarine Cortex | 1078 | 44.83 | 26.16 | 40.21 | 2.72 |
| Lateral Occipital Cortex, inferior division | 86 | 41.67 | 27.08 | 42.13 | 2.60 |
| Lateral Occipital Cortex, superior division | 2279 | 46.82 | 22.63 | 50.20 | 2.78 |
| Left Cerebral White Matter | 1223 | 54.20 | 33.90 | 44.27 | 2.68 |
| Left Hippocampus | 24 | 56.92 | 49.67 | 29.38 | 2.51 |
| Lingual Gyrus | 1247 | 45.61 | 31.36 | 33.60 | 2.61 |
| Middle Frontal Gyrus | 122 | 31.47 | 76.63 | 53.34 | 2.76 |
| Middle Temporal Gyrus, anterior division | 13 | 21.46 | 63.38 | 22.77 | 2.76 |
| Middle Temporal Gyrus, posterior division | 112 | 19.11 | 53.76 | 28.85 | 2.63 |
| Occipital Fusiform Gyrus | 90 | 50.72 | 22.52 | 27.84 | 2.58 |
| Occipital Pole | 1446 | 46.26 | 16.54 | 47.23 | 2.80 |
| Paracingulate Gyrus | 585 | 44.03 | 80.84 | 49.78 | 2.66 |
| Parahippocampal Gyrus | 227 | 50.81 | 46.42 | 29.16 | 2.63 |
| Planum Polare | 54 | 24.43 | 60.59 | 27.48 | 2.54 |
| Postcentral Gyrus | 81 | 53.31 | 41.80 | 61.38 | 2.79 |
| Precuneous Cortex | 1985 | 45.34 | 34.89 | 58.11 | 2.81 |
| Right Thalamus | 22 | 38.09 | 51.32 | 33.36 | 2.68 |
| Subcallosal Cortex | 137 | 47.45 | 72.78 | 29.40 | 2.69 |
| Superior Frontal Gyrus | 641 | 38.11 | 72.13 | 65.46 | 2.66 |
| Superior Parietal Lobule | 26 | 52.81 | 36.92 | 65.15 | 2.58 |
| Superior Temporal Gyrus, anterior division | 11 | 21.64 | 63.64 | 24.64 | 2.69 |
| Superior Temporal Gyrus, posterior division | 39 | 20.44 | 53.62 | 32.15 | 2.56 |
| Supracalcarine Cortex | 176 | 42.75 | 29.20 | 43.55 | 2.83 |
| Temporal Fusiform Cortex | 69 | 53.84 | 45.33 | 22.84 | 2.60 |
| Temporal Occipital Fusiform Cortex | 262 | 49.58 | 37.13 | 28.96 | 2.75 |
| Temporal Pole | 308 | 53.31 | 70.55 | 21.70 | 2.66 |
